# Evaluation of an immunochromatographic point-of-care test for the detection of failure of transfer of passive immunity in calves

**DOI:** 10.1186/s13028-023-00707-9

**Published:** 2023-09-28

**Authors:** Christina Hartsleben, Katharina Lichtmannsperger, Alexander Tichy, Nicole Hechenberger, Thomas Wittek

**Affiliations:** 1 Department for Farm Animals and Veterinary Public Health, University Clinic for Ruminants, University of Veterinary Medicine Vienna, Veterinaerplatz 1, Vienna, 1210 Austria; 2 Bioinformatics and Biostatistics Plattform, University of Veterinary Medicine Vienna, Veterinaerplatz 1, Vienna, 1210 Austria; 3 Animal Health Service Salzburg, Bundesstraße 6, Wals-Siezenheim, 5071 Austria

**Keywords:** Brix, Calf-side test, Colostrum, FASTest® IgG bovine, IgG

## Abstract

**Background:**

As calves are born without circulating immunoglobulin G (IgG) they depend on transfer of passive immunity via colostrum within the first hours of life. If calves are not sufficiently supplied with high qualitative colostrum they suffer from Failure of Transfer of Passive Immunity (FTPI). The objectives of this study were to evaluate a calf-side point-of-care test to detect calves with FTPI and to evaluate the cut-offs for a positive test result. Two hundred fifty calves from 11 dairy farms (born between September 2021 and September 2022) were included, whereof 23 were excluded due to incomplete data. Twelve to 16 h *post partum* the farmers carried out a point-of-care test (FASTest® IgG bovine, Megacor, Austria) using a whole blood sample. Between the 3rd and the 6th day of age, all calves were physically examined and blood samples were collected to carry out further point-of-care tests using whole blood supernatant and plasma and for measuring the Brix values in serum and plasma. Brix values in serum were used as reference for the evaluation of the point-of-care test between the 3rd and the 6th day of age, as radial immunodiffusion assays could not be conducted simultaneously.

**Results:**

Brix values were not normally distributed (median at 8.6% and 9.3% in serum and plasma). In this study, the cut-off values for the point-of-care tests using whole blood supernatant and plasma were at 8.3% Brix in serum. FASTest® IgG bovine shows high sensitivities of 90% and 84% and specificities of 70% and 72% for whole blood supernatant and plasma.

**Conclusions:**

Of the 227 investigated calves, 39.7% showed Brix values of < 8.4% (cut-off for FTPI) which indicates an urgent need to improve colostrum management. The results of the study suggest that the FASTest® IgG bovine is a suitable on-farm method to assess FTPI in whole blood supernatant and plasma of calves between the 3rd and the 6th day of age. However, the results also show that FASTest® IgG bovine is not adequate to test for FTPI using whole blood at 12 to 16 h *post partum*.

## Background

Due to the cotyledonary synepitheliochorial structure of the bovine placenta, calves are not supplied with maternal antibodies during pregnancy and consequently they are born without circulating immunoglobulin G (IgG) [1, 2]. Therefore, calves rely on transfer of passive immunity with IgGs (~ 85–90% IgG1) from maternal colostrum which has to be provided during the first hours of life [1, 2]. Neonatal calves that fail to absorb sufficient colostral IgG therefore suffer from Failure of Transfer of Passive Immunity (FTPI). The threshold value for FTPI varies in literature. Many sources give a serum IgG concentration below 10 mg/mL as the cut-off value [3, 4]. Others, however, raise the cut-off value up to a serum IgG concentration of 15 mg/mL [5]. FTPI is known as a significant problem that can lead to early calf losses due to gastroenteritis, pneumonia or septicaemia [3, 6, 7]. Besides high economic losses and reduced profitability, the increased morbidity and mortality rates pose a major animal welfare issue [8–12]. A study from Switzerland investigated 373 dam-calf pairs, whereof 162 (43.5%) of the calves showed FTPI [13]. It has been reported that the probability of a low serum immunoglobulin concentration in calves increases significantly (odds ratio = 10.7), if the colostrum contains less than 50 g/L IgG [13]. Preliminary results from a project by the Austrian Animal Health Service on the evaluation of colostrum management on dairy farms show that 49.8% of the investigated colostrum samples showed Brix values of less than 22% [14]. Besides high colostrum quality the time between parturition and colostrum delivery to the neonatal calf plays an essential role, since the absorption of immunoglobulins in the intestine decreases within the first 24 h of life and completely ceases at 24 to 36 h. In summary, timing of colostrum feeding after parturition (within 2 h), colostrum quality (≥ 50 g/L IgG) and the amount of colostrum fed to the calf (> 4 L) within the first hours of life are the most important factors to prevent FTPI [1, 3, 15–17]. Furthermore, the efficiency of immunoglobulin absorption tends to be higher in colostrum with low bacterial contamination [18]. In an Austrian online questionnaire on calf management, only 20.8% of the farmers had a colostrum testing protocol, and of these, 86.1% based the protocol on visual inspection [19]. It is essential for farmers to be able to evaluate the success of passive immunity transfer at the herd-level to improve their colostrum management [20, 21]. Although there are various direct methods to assess the immunoglobulin concentration in calves they are rarely used in practice since they are typically time consuming and expensive. The radial immunodiffusion (RID) assay is the gold standard method to measure the quantity of IgG in calf serum [3, 22]. This method has to be performed by laboratory technicians and takes 18 to 24 h. A common alternative is to use indirect methods such as the Brix refractometer using calf plasma or serum [23]. Since the correlation between the measurement by Brix refractometry and the RID is good (r = 0.93) [23], Brix refractometry can be considered a reliable method to directly identify FTPI under field conditions [24–26] by measuring the total solids which approximates to the total protein concentration. The serum Brix measurements of the calves can be categorized using the thresholds described elsewhere [1, 23, 26–29]. Cut-off values to detect FTPI by measuring Brix percentage in serum vary from 7.8% [26, 28], 7.9% [29], 8.4% [23] to 8.7% [27]. Because of these variations, the Brix measurements can be divided into the four categories “excellent” (Brix level ≥ 9.4%), “good” (8.9–9.3%), “fair” (8.1–8.8%) and “poor” (< 8.1%) [1], which can also be used on a herd-level. Blood samples are obviously required to assess the immunoglobulin concentration in serum or plasma. In Austria taking blood samples puncturing a vein is restricted to veterinarians but farmers are allowed to scarify the skin producing a blood drop from capillaries. Therefore, indirect methods such as the Brix refractometer are rarely used in practice. It is frequently not practical to take a venous blood sample in the first hours of the calf’s life. However, a commercially available point-of-care test could be used to assess immunoglobulin concentrations by farmers since only a few microliters of blood are required for the procedure. To the best of our knowledge, there has been limited research been done on testing the transfer of passive immunity of neonatal calves 12 to 16 h after birth [30]. In the majority of studies, calves from 24 h of age until 7 [31], 8 [32], 11 [33] or 15 days of age [34] were studied. To implement a reliable tool to assess the IgG status of the neonatal calf, an early, inexpensive and practical tool such as the FASTest^®^ IgG bovine (FASTest^®^ IgG bovine, Megacor, Austria) might be used. The point-of-care test is a qualitative test for the detection of bovine IgG in serum, plasma or whole blood supernatant. According to the manufacturer’s specifications, the point-of-care test shows a negative result if the immunoglobulin concentration is less than 10 mg/mL and the FASTest^®^ IgG bovine is licensed to be used in calves from 24 h up to 7 days of life.

The objectives of this study were to evaluate the feasibility of a calf-side point-of-care test carried out at different times *post partum* (*pp*) to detect calves with FTPI and to evaluate the cut-offs for a positive test result. Since the point-of-care test is not approved for whole blood, the results with this medium were assessed by comparing with the results of point-of-care tests carried out with whole blood supernatant and plasma.

We hypothesized (1) That the threshold of the point-of-care test to indicate FTPI is 8.4% Brix in serum (2) That the results of the point-of-care tests carried out 12 to 16 h *pp* and the point-of-care test carried out 3 to 6 days *pp* are strongly associated indicating that the early measurement has a sufficient diagnostic value. Therefore, the results of the point-of-care tests with whole blood, whole blood supernatant and plasma were compared.

## Methods

### Ethical consideration

This study was approved by the Ethics and Animal Welfare Committee (ETK) of the University of Veterinary Medicine, Vienna and the Austrian national authorities, according to § 26 of the Tierversuchsgesetz 2012 – TVG 2012 (GZ.: 2021 − 0.644.875).

### Study farms and animals

**Fig. 1:**
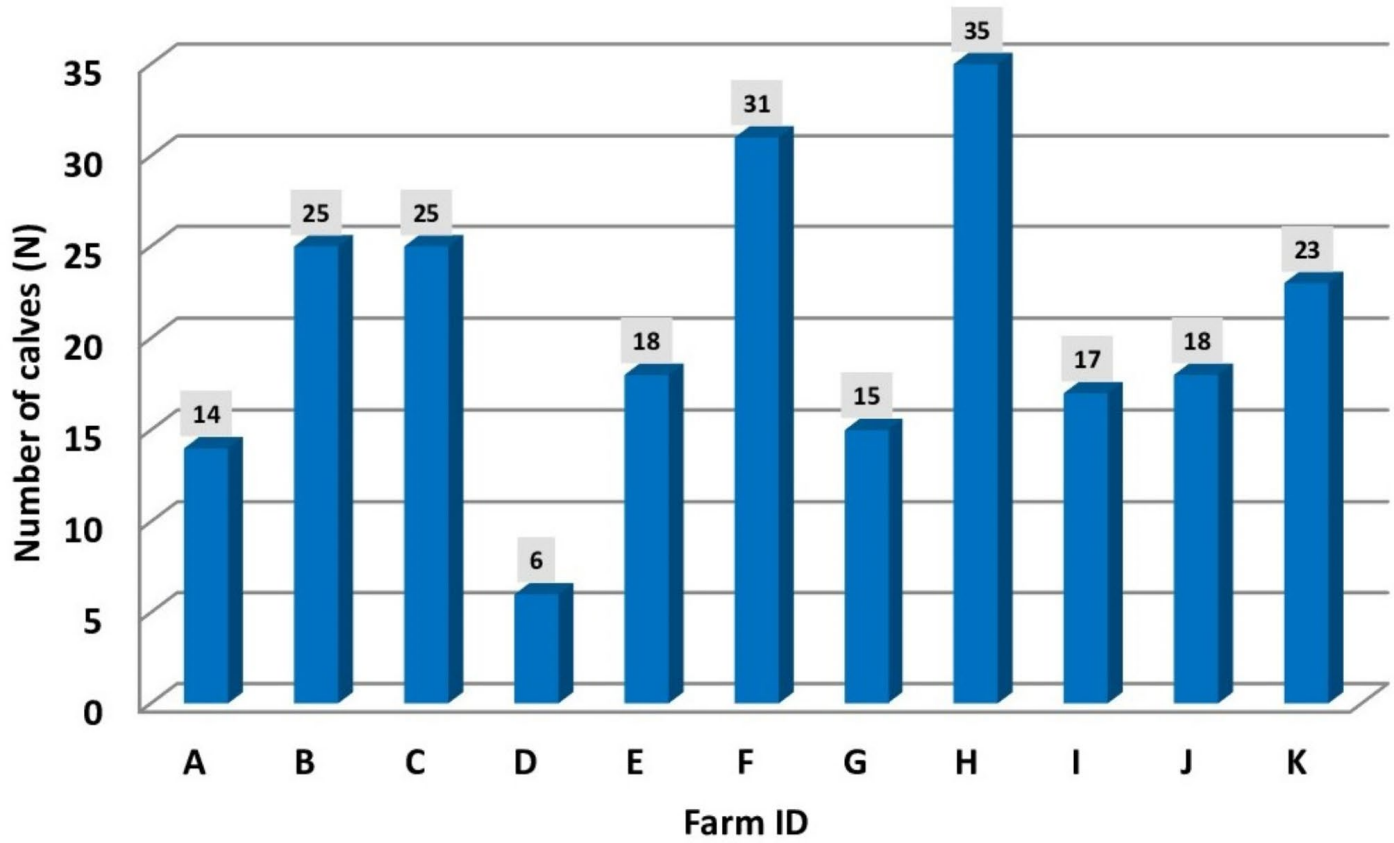
Total number of included male and female calves per farm. In total, 227 calves originating from 11 dairy farms participated in the study

Two hundred and fifty calves from 11 dairy farms in the region of Enns-Pongau and Lungau (federal state of Salzburg, Austria) born between September 2021 and September 2022 were included (see Fig. 1). The calves were included in the study on the sequence of their birth and excluded only, if they were extremely stressed, uncooperative or died within the first 6 days of life (n = 0). Seven farms already participated actively in a previous project by the Austrian Animal Health Service on the evaluation of colostrum management in dairy farms. A part of this larger investigation has been published previously [14]. The remaining 4 farms joined the study on their own intention as they could provide additional samples conveniently. In total, 227 female and male calves aged up to 6 days of age were finally involved after removing the data of 23 calves due to missing information. The breed was primarily Fleckvieh (Simmental) (n = 118 with 59.3% female and 40.7% male calves), Pinzgauer (n = 65 with 33.8% female and 66.2% male calves) and cross-breeds (Belgian Blue) (n = 44 with 45.5% female and 54.5% male calves). All calves were separately from their dams within one hour and the farmers took care that suckling was not possible. Of the 227 calves included, 217 calves received colostrum from their own dam and 10 calves received non-maternal colostrum (pooled frozen colostrum, fresh colostrum from another cow than mother). Two hundred and eight calves received their first meal within 4 h *pp*. Nineteen calves received the colostrum after ≥ 4 h. Of these, 17 calves initially showed no suckling reflex and 2 calves were fed later since the farmer did not manage to deliver the colostrum within 4 h. One hundred and eighty-seven calves had a colostrum intake of ≥ 2 L whereas 40 calves had a colostrum intake of less than two liters. All calves were in barns and they only received colostrum and transition milk within the first 6 days *pp*. The calves were fed twice daily under supervision of the farmers. Twelve calves were fed several times a day because their general condition and sucking reflex were poor. Factors that were associated with colostrum quality, FTPI and their impact on health events in the first three weeks of life was also evaluated and already published [35].

### Point-of-care test implementation at 12 to 16 h post partum by farmers

**Fig. 2:**
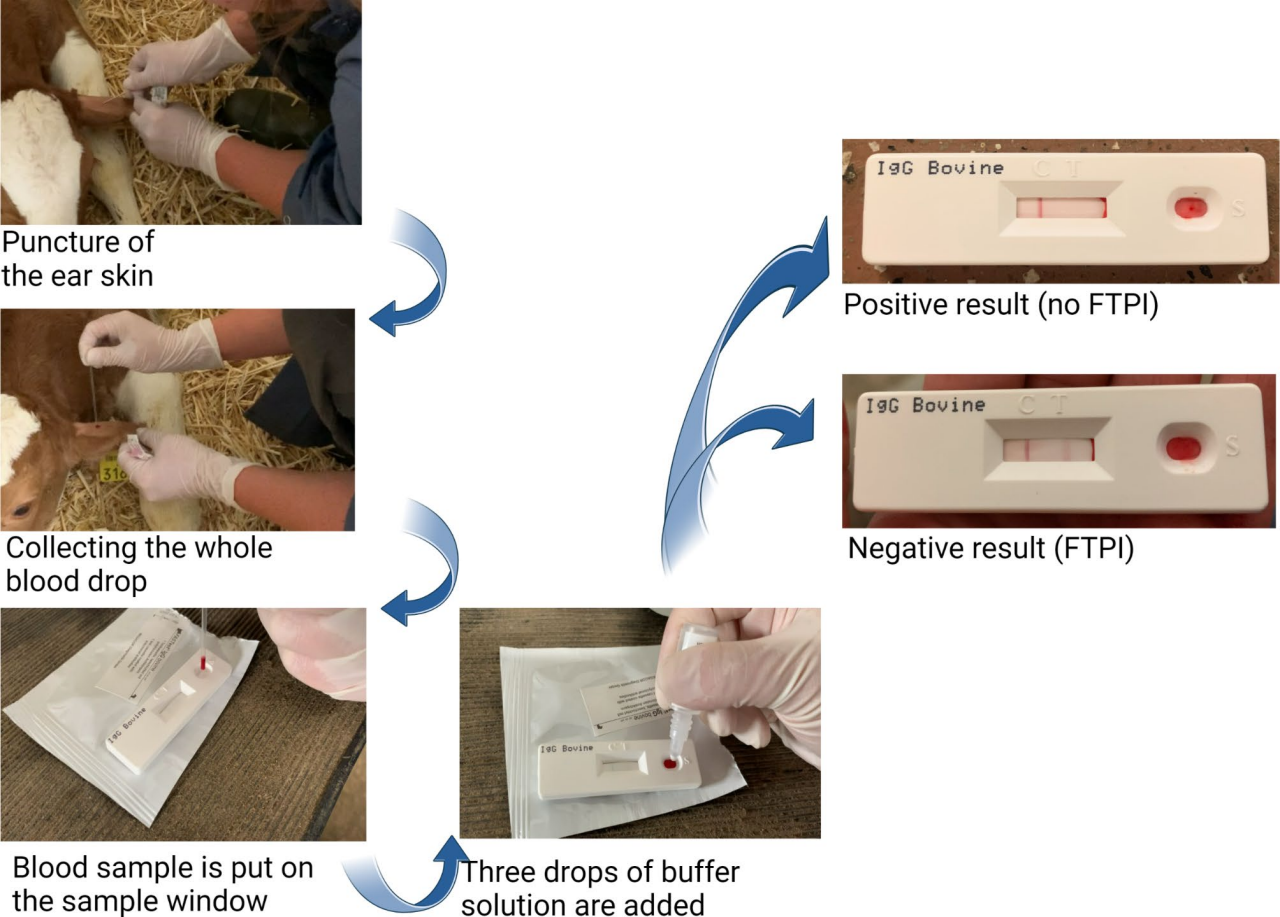
All 11 farms were visited by the principal author and the farmers received a training on the usage of the point-of-care test (FASTest® IgG bovine, Megacor, Austria) for the detection of Failure of Transfer of Passive Immunity (FTPI). The farmers draw the samples 12 to 16 h *post partum*

Prior to the study, all 11 dairy farms were visited by one author (CH) and the farmers were trained using the point-of-care test system (FASTest® IgG bovine, Megacor, Austria) (Fig. 2). The FASTest® IgG bovine is a qualitative test for the detection of bovine IgG in serum, plasma or whole blood supernatant. The point-of-care test includes a control line and a test line. According to the manufacturer’s specifications, the test line solely appears, if the immunoglobulin content is less than 10 mg/mL (FTPI). Twelve to 16 h *pp*, the point-of-care test was carried out by the farmers as described (Fig. 2). The skin of right or left ear edge was scarified using a hypodermic needle. Subsequently, the whole blood drop (approximately 20 µL) was collected using a plastic pipette and the test was carried out according to the manufacturers specifications. Briefly, the blood sample was put on the sample window of the test cassette and three drops of buffer solution (approx. 120 to 150 µL) were added to the sample window. After 10 min at 20 to 25 °C the test kit was read by the farmers. The test results were recorded on paper and a digital photograph was taken of each test result. Subsequently, the photograph was sent to the principal author (CH) for further review of the test result. It is common to feed transition milk of the mother to the calves for 3 days. Farmers were instructed to carry on with their herd-specific management regardless of the point-of-care test result.

### Point-of-care test implementation at 3 to 6 days of age by the principal author (CH)

**Fig. 3:**
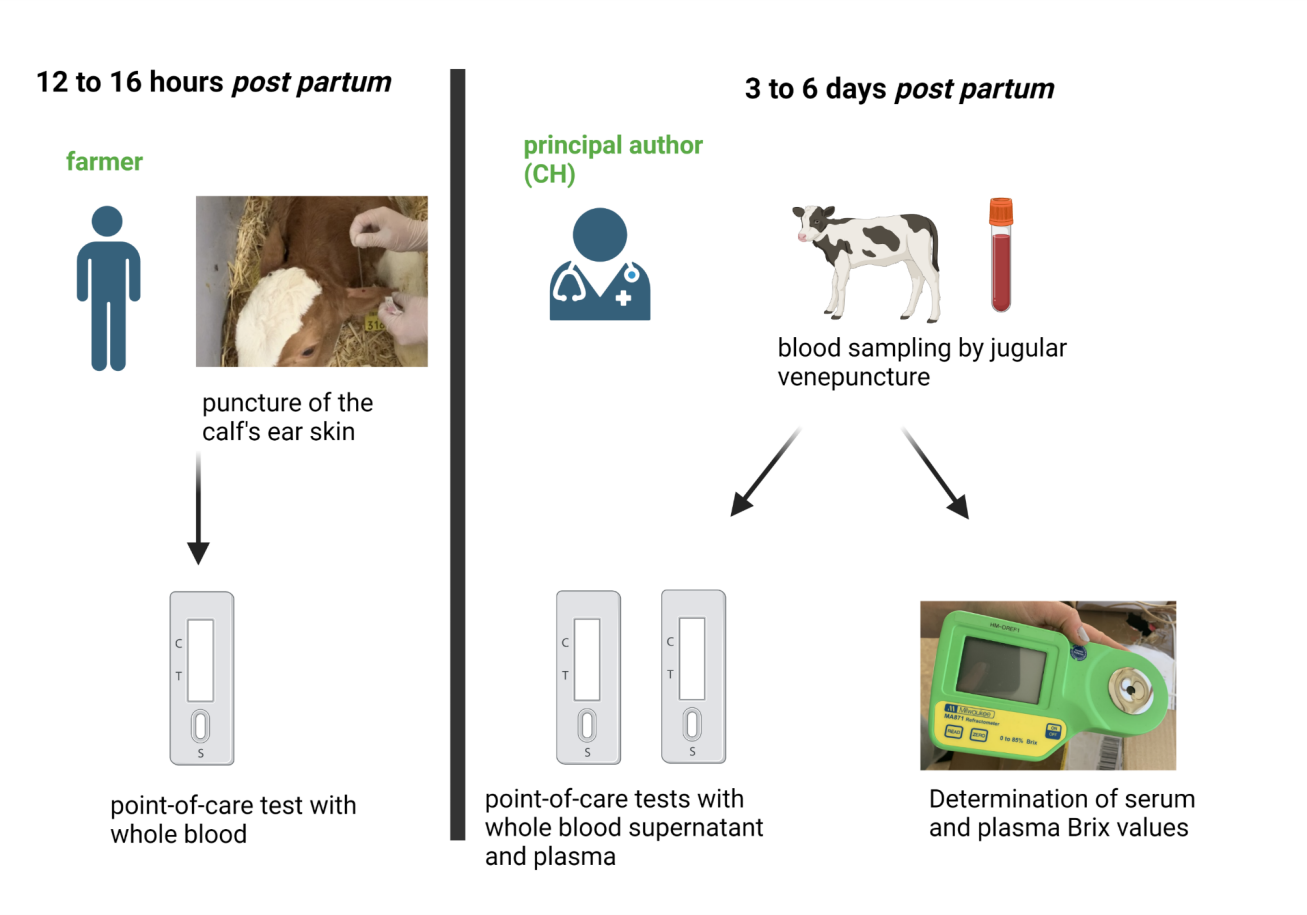
The samples were collected 12 to 16 h *post partum* (whole blood) by the farmers and 3 to 6 days *pp* (EDTA and serum) by the principal author (CH). All calves were clinically examined at the time of sampling. The figure illustrates the steps from sample collection to sample analysis

EDTA and serum samples were collected by the principal author (CH) from calves between 3rd and 6th day of age by jugular venipuncture using an 18-gauge needle and vacutainer tubes (Vacuette®, Greiner Bio-One GmbH, Austria). The point-of-care tests were performed using two different samples: whole blood supernatant and plasma. To receive whole blood supernatant, EDTA blood samples were left untouched in an upright position for 5 min at 20 to 25 °C. Plasma and serum samples were produced by centrifugation at 1,500 g for 10 min at 20 to 25 °C on the farms (CGOLDENWALL 800D Electric Centrifuge Medical Lab Centrifuge 4,000 rpm with CE 6 × 20 mL, Zhengzhou Jin Chen Electronic Technology Co. Ltd., China). All tests were carried out according to the manufacturer’s specifications under field conditions (Fig. 3).

### Reference method: brix refractometry

Serum and plasma Brix values were determined immediately after sample collection under field conditions (approx. 20 to 25 °C) on each farm using a digital Brix refractometer (MA871 Refractometer, Hebesberger, Austria) (Fig. 3). The Brix refractometer (0 to 85% Brix) was calibrated using deionized water. Calibration was carried out routinely at the beginning of the analysis on each farm and following the measurement of 10 serum or plasma samples. After calibration, serum or plasma was pipetted onto the prism using a one-way 2 ml plastic pipette. The Brix percentage was recorded twice and the mean value was used for statistical analysis. Aliquots were made in 1.5 mL Eppendorf tubes® (Eppendorf®, Eppendorf Austria GmbH, Austria) and transported on ice in a polystyrene box. All samples were frozen within two hours at minus 18 °C.

### Statistical analysis

Descriptive and explorative statistical analysis was performed using Microsoft Excel 2010 (Microsoft®, Washington, USA) and IMB® SPSS® Statistics Version 28 (IBM®, New York, USA). The serum and plasma Brix percentages of the calves were categorized using the threshold described elsewhere (excellent = ≥ 9.4%, good = 8.9–9.3%, fair = 8.1–8.8%, poor = < 8.1%) [1]. For each calf three point-of-care tests were performed: One by the farmer (whole blood) and two by the principal author (whole blood supernatant and plasma) (see Fig. 3). Statistical analysis was performed in two stages: Primary, test validity of point-of-care tests with whole blood supernatant and plasma using the BRIX values in serum as reference was assessed using the Youden-Index and ROC-analysis. The Youden index, which is calculated from the sum of sensitivity and specificity minus 1, to calculate the optimum limit value. The area under the receiver operating characteristic (ROC) curve (AUC) was included as a measure of the quality of the test. The AUC values range between 0.5 and 1.0, where the higher values indicate better quality. Second, the results of the three different point-of-care tests for each individual calf were compared to each other. There were three comparisons: Firstly, the comparison of the results of the farmer’s point-of-care tests using whole blood with the results of the point-of-care tests with whole blood supernatant, carried out by the principal author. Secondly, the results of the farmer’s point-of-care tests using whole blood were compared to the results of the point-of-care tests with plasma. Thirdly, the results of the point-of-care tests with whole blood supernatant were compared to those with plasma (both carried out by the principal author). Definition of a negative point-of-care test result was if the calf showed FTPI (IgG less than 10 mg/mL according to the manufacturer’s specifications which equals 8.4% Brix in serum according to previous investigations [23]). For this purpose, cross tables were created and the Cohen’s Kappa (κ) was used. Cohen’s Kappa was calculated based on the comparison of point-of-care tests and describes the agreement between them. Kappa values can range from 0 to 1 and they are interpreted as follows: ≥0.81 very good agreement; 0.61 to 0.8 good agreement; 0.41 to 0.6 moderate agreement; 0.21 to 0.4 fair agreement; and ≤ 0.2 poor agreement [36]. Using cross tables, sensitivity and specificity of the point-of-care test comparisons were calculated. All tests were calculated with a significance level of P < 0.05. Tests of normality were carried out using Kolmogorov-Smirnov test.

## Results

### Farms and calves

**Fig. 4:**
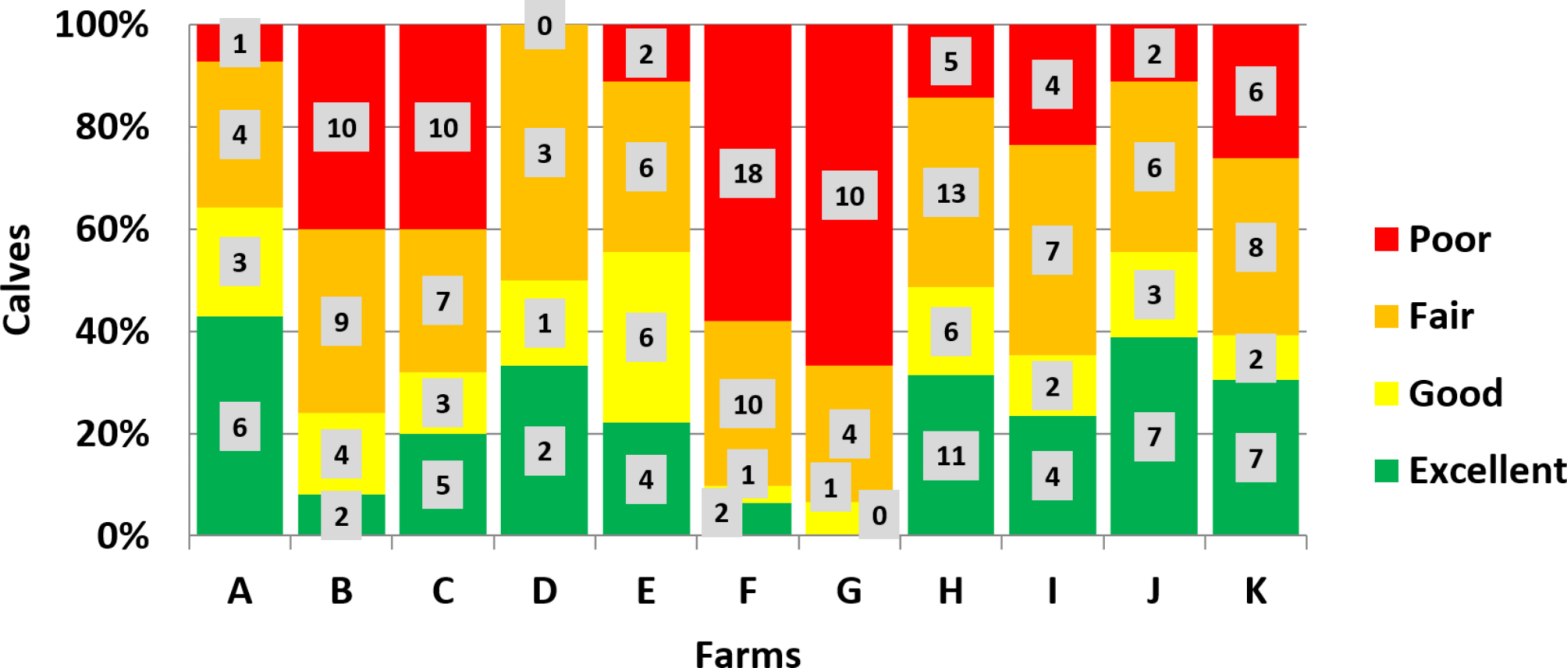
Summary of the four categories for immunoglobulin G levels based on the BRIX measurements in serum on the 11 included farms according to Godden and colleagues (2019)

In total 227 calves originating from 11 farms were included. IgG levels based on the measurements of the calves’ serum were categorized as poor, fair, good and excellent [23] (Fig. 4). The proportion of calves that had low levels of IgG varied greatly across the farms.

### Brix values

The intra- and inter-reliabitity for the digital Brix refractometry was not calculated in the present study as the accurate assessment [37]. According to the Kolmogorov-Smirnov-test, P-value for Brix values in serum was < 0.001 and for Brix values in plasma 0.002, which means that data were not normally distributed. Regarding the Brix values in serum, there was a minimum of 6.3% and a maximum of 11.8%. Percentiles (10th, 25th, 75th, 90th ) were at 7.6%, 8.0%, 9.3% and 9.9% respectively. The median was at 8.6% with a variance of 0.9%. For Brix values in plasma the minimum was at 7.0% and the maximum at 12.4%. The percentiles in this case were at 8.4%, 8.7%, 9.3%, 9.9% and 10.5%. A median of 9.3% with a variance of 0.8% was calculated.

**Fig. 5:**
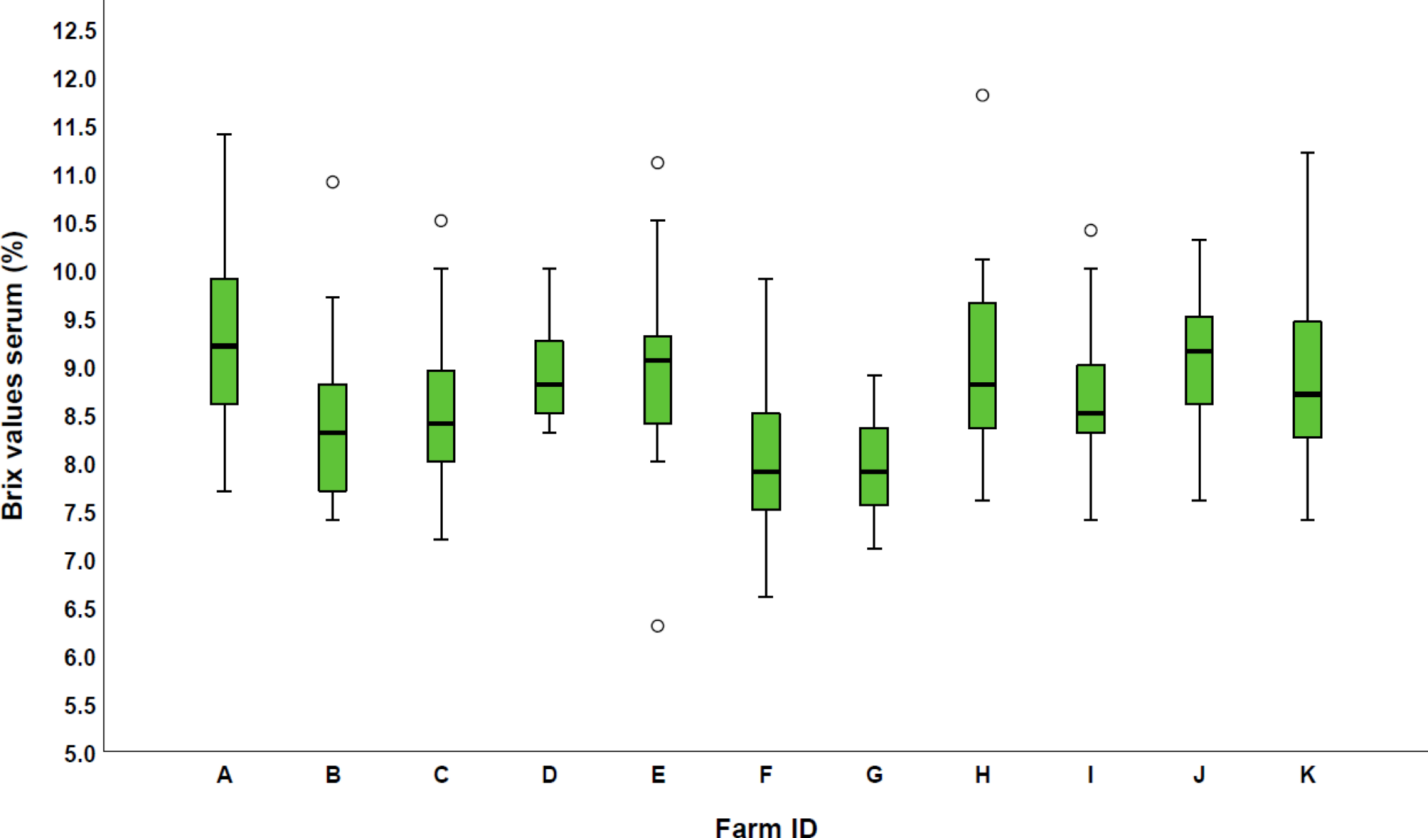
Boxplots of the Brix values in serum for each individual farm (n = 11) and 227 investigated calves

In total, 137 of the calves (60.3%) and 90 of the calves (39.7%) showed serum Brix values of ≥ 8.4% and serum Brix values of < 8.4%, respectively (Fig. 5). Consequently, 39.7% of the calves were classified as suffering from FTPI. The statistical measures varied between the individual farms. Further details are published elsewhere [35].

### Receiver operating curves

#### Evaluating cut-offs for the point-of-care test carried out by the principal author using whole blood supernatant

**Fig. 6:**
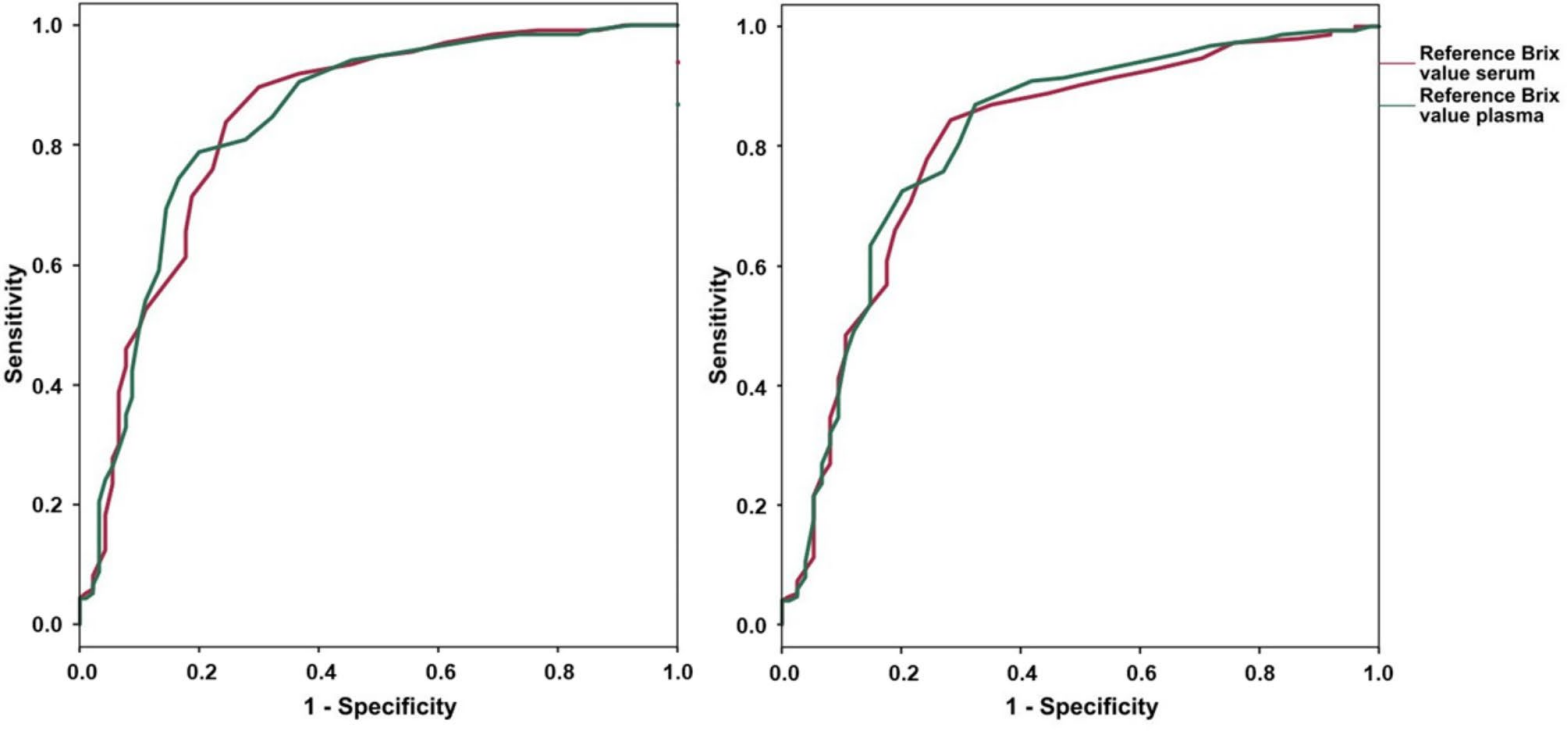
Receiver Operating Curve for the point-of-care test (FASTest® IgG bovine, Megacor) using whole blood supernatant (left) and plasma (right) carried out 3 to 6 days *post partum*. The Brix values from serum and plasma were used as reference to calculate the optimal threshold

The point-of-care test using whole blood supernatant carried out by the principal author between 3rd and 6th day *pp* was carried out using 137 ‘no FTPI’ and 90 FTPI samples. In this case, the AUC was 0.84 and 0.88 when using the Brix values from serum and the Brix values from plasma (collected between the 3rd and the 6th day of age). The optimal cut-off was set at 8.3% Brix for serum (SE = 0.90; SP = 0.70) and 9.2% Brix for plasma (SE = 0.79; SP = 0.80). The Youden Index was 0.60 and 0.59 for serum and plasma, respectively (Fig. 6).

#### Evaluating cut-offs for the point-of-care test carried out by the principal author using plasma

The point-of-care test using plasma carried out by principal author between 3rd and 6th day *pp* was carried out on 153 ‘no FTPI’ and 74 FTPI test results. The AUC was 0.81 and 0.81 when using the Brix values from serum and the Brix values from plasma (collected between the 3rd and the 6th day of age), respectively. The optimal cut-off was set at 8.3% Brix for serum (SE = 0.84; SP = 0.72) and 8.9% Brix for plasma (SE = 0.87; SP = 0.68). The Youden Index was 0.56 and 0.55 for serum and plasma, respectively (Fig. 6).

### Point-of-care test comparison

In order to know if the point-of-care test 12 to 16 h *pp* and the point-of-care tests between 3rd and 6th day *pp* are strongly associated the results of all 3 point-of-care tests were compared to each other (Table 1).

**Table 1:** Comparison of the three point-of-care tests carried out for each individual calf

Point-of-care test	Comparison test	PPV (95% CI)	NPV (95% CI)	SE (95% CI)	SP (95% CI)	К (95% CI)
Whole blood*	whole blood supernatant#	71.4% (64.6%; 77.5%)	88.1% (76.1%; 95.6%)	96.4% (92.3%; 98.7%)	41.1% (31.3%; 51.4%)	0.412 (0.300; 0.525)
Whole blood*	plasma#	76.2% (69.7%; 82.0%)	71.4% (56.8%; 83.5%)	92.2% (87.2%; 95.7%)	40.5% (29.8%; 51.9%)	0.368 (0.240; 0.496)
Whole blood supernatant#	plasma#	98.5% (95.6%; 99.8%)	80.0% (71.0%; 87.4%)	88.2% (82.5%; 92.7%)	97.3% (91.9%; 99.5%)	0.81 (0.732; 0.889)

This table gives an overview of the comparison of the three point-of-care tests carried out for each individual calf. As there was no RID implemented, the three point-of-care tests were compared to each other in order to calculate these values. Positive predictive value (PPV), negative predictive value (NPV), sensitivity (SE), specificity (SP) and Cohen’s Kappa (К) of one point-of-care test (FASTest® IgG bovine, Megacor) using different blood collecting time points *pp* (*12 to 16 h *pp* by the farmers; #3 to 6 days *pp* by the principal author) and different media. The lower and upper 95% confidence intervals are given in parentheses (95%CI).

#### Comparison of point-of-care test results from whole blood (12 to 16 h post partum) and whole blood supernatant (3 to 6 days post partum)

Of the 227 included calves 132 calves (60.4%) had a sufficient immunoglobulin concentration and 90 calves (39.6%) had an insufficient immunoglobulin concentration according to the test result after 3 to 6 days using the whole blood supernatant samples. Of the 185 (100%) point-of-care test results showing ‘no FTPI’ gained after 12 to 16 h by the farmer, 132 (71.4%) were correctly identified as ‘no FTPI’ and 53 (28.6%) were incorrectly identified as ‘no FTPI’ (Table 2). The sensitivity and specificity in this comparison were 96.4% and 41.1%, respectively. The kappa coefficient was 0.412 (0.300; 0.525), indicating a moderate agreement (see Table 1).

**Table 2:** Cross tabulation on the point-of-care test results

		FASTest^®^ IgG bovine with whole blood supernatant (3 to 6 days *p.p.*)		
		no FTPI	FTPI	total
**FASTest** ^**®**^ **IgG bovine with whole blood** **(12 to 16 h** ***p.p.*** **)**	no FTPI	132	53	185
	FTPI	5	37	42
	total	137	90	227
		**FASTest** ^**®**^ **IgG bovine with plasma (3 to 6 days** ***p.p.*** **)**		
		no FTPI	FTPI	total
**FASTest** ^**®**^ **IgG bovine with whole blood** **(12–16 h** ***p.p.*** **)**	no FTPI	141	44	185
	FTPI	12	30	42
	total	153	74	227
		**FASTest** ^**®**^ **IgG bovine with plasma (3–6 days** ***p.p.*** **)**		
		no FTPI	FTPI	total
**FASTest** ^**®**^ **IgG bovine with whole blood supernatant** **(3–6 days** ***p.p.*** **)**	no FTPI	135	2	137
	FTPI	18	72	90
	total	153	74	227

This table shows a cross tabulation on the point-of-care test results carried out by the farmers (12 to 16 h *post partum (pp)*) and by the principal author (3 to 6 days *pp*) using different media for the test

#### Comparison of point-of-care test results from whole blood (12 to 16 h post partum) and plasma (3 to 6 days post partum)

Of the 227 included calves, 153 calves (67.4%) showed ‘no FTPI’ and 74 calves (32.6%) had an insufficient immunoglobulin concentration according to the test result after 3 to 6 days using the plasma samples. Of the 185 (100%) point-of-care test results gained after 12 to 16 h by the farmer, 141 (76.2%) were correctly identified ‘no FTPI’ and 44 (23.8%) were incorrectly identified as ‘no FTPI’ (Table 2). The sensitivity and specificity in this comparison were 92.2% and 40.5%, respectively. The kappa coefficient was 0.368 (0.240; 0.496), indicating a fair agreement (see Table 1).

#### Comparison of point-of-care test results from whole blood supernatant and plasma (3 to 6 days post partum)

As described in the manufacturer’s specifications, the point-of-care test using plasma after 3 to 6 days *pp* was implemented as the reference test for comparison. Two hundred and twenty seven calves were included. Of these calves, 137 calves showed ’no FTPI’ and 90 calves were categorized as having FTPI, according to the point-of-care test with whole blood supernatant. Of the 137 calves that showed ”no FTPI”, 135 were correctly and 2 were incorrectly identified as having ”no FTPI” in comparison to the point-of-care test with plasma (Table 2). The sensitivity and specificity in this comparison were 88.2% and 97.3%. The kappa coefficient was 0.81 (0.732; 0.889), indicating a good agreement (Table 1).

## Discussion

### Occurrence of FTPI

In order to protect calves against FTPI, adequate supply of high qualitative colostrum is necessary. Calf-side point-of-care tests would be a suitable on-farm method to detect calves with FTPI with some limitations. According to the manufacturer’s specifications of FASTest® IgG bovine, the cut-off value is set at 10 mg/mL which has been described to equal 8.4% Brix in serum with a sensitivity and specificity of 88.9% [23]. Different Brix values have been estimated to correspond to a serum IgG concentration of 10 mg/mL [1, 23, 26–29]. It has to be stressed that studies have used different reference tests to determine an optimal cut-off. For example, serum total protein (STP) or enzyme-linked immunosorbent assays are used as comparison methods to implement a value corresponding a serum IgG concentration of 10 mg/mL [26]. RID as gold standard would be optimal as a reference comparison [23]. Therefore, we hypothesized that the threshold of the point-of-care test indicating FTPI is 8.4% in serum [23], as it has been described that a serum IgG concentration of 10 mg/mL equals 8.4% Brix in serum with a high sensitivity and specificity [23]. Furthermore, there was a high correlation of Brix percentage and IgG (analyzed by RID) of r = 0.93 [23]. In general, it has to be emphasized, that one single cut-off value is not adequate to categorize calves with FTPI on a herd-level. It would be beneficial to divide the serum brix values of the calves into categories, for instance as described by Godden and coworkers [1]. A dichotomous approach (FTPI yes or no) is possible for instance by using such a point-of-care test. However, on a herd-level the categorization would be beneficial since it should be emphasized that calves do not just have an IgG level of > 10 mg/mL, they should have an excellent colostrum supply with > 25 mg/mL. In the present study, however, there had to be a limit value based on the validity of the point-of-care test (yes/no).

This applies not only to the cut-off value determination of Brix percentage, but also to the cut-off value determination of other units, such as serum IgG concentration. Regarding this, cut-off values range from 10 mg/mL [3, 4] to 15 mg/mL [5]. Further studies are needed to clarify this issue.

### Brix percentages and their cut-off values

In total, 90 calves (39.7%) showed serum Brix percentages of < 8.4%, respectively. In other studies, the number of calves with FTPI was at 27% [28], 13% [26], 8.3% [29], 4.75% [23] and 43.3% [27]. These great differences are due on the one hand to the different cut-offs, and on the other hand, there are both, geographical- and management-related differences. Furthermore, parameters such as season, temperature, time of sampling and cattle breed can have an effect on the results. In the present study, there are differences between the individual farms. Further studies and a greater study population are necessary to show the impact of these parameters on Brix values.

Brix cut-off values in serum were at 8.3% for point-of-care tests using plasma and whole blood supernatant. According to the ROC analysis, sensitivity and specificity were at 84.3% and 71.6% for point-of-care tests with plasma and at 89.8% and 70.0% for point-of-care tests with whole blood supernatant, respectively. Compared to an investigation carried out on dairy calves, the calculated cut-off values of the present study were lower, possibly explaining the high number of false results (‘no FTPI’) [23]. Assuming the calculated cut-off values in the point-of-care test were higher, more calves would show FTPI, as discussed elsewhere [5].

Brix cut-off values in plasma were also investigated. The cut-offs in this respect were at 8.9% for point-of-care tests with plasma and 9.2% for point-of-care tests with whole blood supernatant, respectively. There are multiple studies showing a substantial difference between serum and plasma Brix levels of 8.7% Brix in serum and 9.4% Brix in plasma and 7.8% Brix in serum and 8.6% Brix in plasma [27, 28]. The cut-off results of the present investigation were within the range of the aforementioned studies. One of the potential explanations for cut-offs in plasma being higher than in serum might be because plasma contains coagulation proteins such as fibrinogen, which is soluble and clots during serum processing [38]. Furthermore, EDTA (used in this study) as well as other anticoagulants (lithium-heparin, citrate or heparin) can incorrectly lead to an increase of plasma total protein concentrations due to an incorrect ratio between blood and the anticoagulant [39].

It has been proven elsewhere that Brix values of serum samples show higher agreement with RID than those from plasma with an accuracy of 79.7% in serum and 74.7% in plasma, respectively [27]. A digital Brix refractometer has been implemented as reference in the present study. Currently, the RID is recognized as gold standard in detecting immunoglobulins in bovine serum samples. Multiple investigations showed that the RID results and the Brix results show a good accuracy. Therefore, it has to be stressed that using the Brix values as reference was suboptimal. With regard to the Brix values in serum on days 3 to 6 of life, 90 calves (39.7%) had FTPI, if a threshold of 8.4% was used [23].

### Point-of-care test comparison

The second hypothesis was that the results of the point-of-care tests carried out 12 to 16 h *pp* and the point-of-care tests carried out 3 to 6 days *pp* give the same test result (FTPI yes or no) indicating that the early measurement has sufficient diagnostic value. Since the point-of-care test is not approved for whole blood, the results with this medium were checked by comparing with the results of point-of-care tests carried out with whole blood supernatant and plasma. It was feasible to carry out the point-of-care test using whole blood from the calf’s ear. Nevertheless, the point-of-care test showed a poor performance in terms of false positive and false negative rates in comparison to the tests carried out after 3 to 6 days of age. It needs to be stressed that no reference test (Brix value) was available for this time point. In future investigations, the gold standard also needs to be carried out in parallel.

In total, 185 calves were identified as having ‘no FTPI’ by the point-of-care test using whole blood after 12 to 16 hours *pp* (by the farmers). Of these 185 calves, 28.7% (53) and 23.8% (44) of the calves had been identified incorrectly using whole blood supernatant and plasma as the reference test for comparison at 3 to 6 days of age. The high number of false ’’no FTPI’ results make the farmers believe that the new-born calves are supplied sufficiently with IgG. The FASTest® IgG bovine has a low specificity 12 to 16 h *pp*, which might be due to any kind of cross reaction between the whole blood cells and the antibodies of the lateral flow ELISA. The exact causes seem to be unknown.

In total, 42 calves have been identified as having FTPI by the farmers’ point-of-care test with whole blood. Of these 42 calves, (11.9%) 5 (comparison test with whole blood supernatant) and 28.6% (12) (comparison test with plasma) had been identified incorrectly. One of the major limitations of the study was, that there was no reference test (digital Brix refractometry) carried out 12 to 16 h after birth. In brief, it can be summarized that the collection of whole blood from the calf’s ear using a capillary was feasible but the timing and the sample type do not seem to be suitable for FASTest® IgG bovine.

Additionally, the qualitative point-of-care test solely divides the calves into the ones having FTPI and the ones not having FTPI. It is well known that the IgG status of the calves should not just be divided dichotomously since there is a difference in morbidity and mortality rates between calves having poor (< 8.1% Brix in serum), fair (8.1–8.8% Brix in serum), good (8.9–9.3% Brix in serum) or excellent (≥ 9.4% Brix in serum) TPI [1]. Therefore, it needs to be further investigated if the point-of-care test is an economically useful investment taking into account the information you receive and the conclusions you can draw from the results.

Point-of-care tests with whole blood supernatant and plasma compared to each other show a very good agreement with a kappa value of 0.81 (ranging from 0.73 to 0.89). The ZAPvet Bovine IgG (ZAPvet Bovine IgG test, ZBx Corp., Toronto, ON, Canada), which is a different calf-side point-of-care test, shows a sensitivity of 82.0% and a specificity of 65.0% [33]. Sensitivity as well as specificity are significantly lower for ZAPvet Bovine IgG than for FASTest® IgG bovine regardless the sample used. FASTest® IgG bovine has a sensitivity of 90% and 84% and a specificity of 70% and 72% for whole blood supernatant and plasma, respectively.

### General limitations

There were some major limitations in the present study. The used reference method was not the gold standard (RID). There is good evidence showing that there is a high correlation between the RID and Brix refractometry [23, 27–29]. Since there is an accuracy of almost 80% between Brix values in serum and RID, this value was used as the reference method [27]. The digital Brix refractometer was used as a fast and cost effective reference method, which can be carried out in the local veterinary practice without any special skills or equipment. Nevertheless, it has to be mentioned that even this method is not cheap. Initial cost may range from $US 200–400 and an annual calibration has to be done. With regard to the calculation of sensitivity and specificity, it has to be emphasized at this point that their calculation would have been preferable using the gold standard method (RID).

As it was not feasible for the principal author (CH) to visit each calf and take a blood sample 12 to 16 h *pp*, due to night parturition for example, farmers were also involved in sample collection. Since the national law allows farmers only to draw capillary blood, whole blood was used even though FASTest® IgG bovine is approved for whole blood supernatant, plasma and serum. According to the manufacture’s specifications, the test time slot for FASTest® IgG bovine is from 24 h up to 7 days *pp*. In future investigations, additional serum and plasma samples have to be drawn from the calves 12–16 h *pp* to describe whether the cross-reactivity of the blood constituents with the antibodies or other factors influence the test results. Another aspect is that the value of IgG may still be increasing at this time point. For example, if a calf did not drink colostrum until 6 h *pp*, there is a high probability of having a test result with FTPI 12 h *pp*. However, since TPI is not yet complete at this time, the same calf might not have FTPI at a later time point of measurement. Another aspect is that providing additional colostrum even 24 h *pp* is beneficial and might increase IgG levels [30, 40]. So, if the point-of-care test had been done at a later time point (for example 18–24 h *post par,tum*), better matches to the results of the tests performed between 3rd and 6th day might have been achieved. Furthermore, Brix refractometry or even better RID should be done at 12 to 16 h *pp* in order to check the presented values for FASTest® IgG bovine at this timeslot.

The 11 farms are a good representation of the region of Enns-Pongau and Lungau (federal state of Salzburg, Austria). In order to get a reliable overview on the actual colostrum supply of calves in the province of Salzburg, further studies are necessary. To the best of our knowledge, this was the first attempt to focus on the feasibility of a calf-side point-of-care test in Austria. Further studies are needed to investigate if capillary whole blood is a reliable medium to test for FTPI in calves at different time points with calf-side point-of-care tests. This study was solely a small scale investigation in a defined region in Austria. To receive data on the true prevalence of FTPI in Austria, further studies are needed including a prior sample size calculation and defined inclusion criteria (randomisation). It is also necessary to do further studies in other countries, because globally, there are many different managements and thus differences in the IgG supply of the calves.

## Conclusions

39.7% of the investigated calves from the regions Enns-Pongau and Lungau, Austria showed Brix values of < 8.4% (cut-off for FTPI). This shows that there is an urgent need for improvement in terms of colostrum management in these specific regions of Austria. This study also determined that the point-of-care tests using whole blood supernatant and plasma carried out between the 3rd and the 6th day of age are suitable to get information on the status of transfer of passive immunity in calves. The point-of-care test showed a sensitivity of 90% and a specificity of 70% for whole blood supernatant and a sensitivity of 84% and a specificity of 72% for plasma using 8.3% Brix as the cut-off. It was feasible to carry out the point-of care test at 12 to 16 h after birth using whole blood collected from the ear edge by the farmer. Further studies are needed to evaluate if an early evaluation of transfer of passive immunity (12 to 16 h *pp*) might be useful and whether the point-of-care test provides an accurate result using whole blood.

## Data Availability

Data available within the article.

## Declarations

### Prior publication

Data have not been published previously.

## References

[1] Godden SM, Lombard JE, Woolums AR (2019). Colostrum management for dairy calves. Vet Clin N Am Food Anim Pract.

[2] Smith VR, Reed RE, Erwin ES (1964). Relation of physiological age to intestinal permeability in the bovine. J Dairy Sci.

[3] Godden SM (2008). Colostrum management for dairy calves. Vet Clin N Am Food Anim Pract.

[4] Vogels Z, Chuck GM, Morton JM (2013). Failure of transfer of passive immunity and agammaglobulinaemia in calves in south-west victorian dairy herds: prevalence and risk factors. Aust Vet J.

[5] Meganck V, Hoflack G, Opsomer G (2014). Advances in prevention and therapy of neonatal dairy calf diarrhea: a systematical review with emphasis on colostrum management and fluid therapy. Acta Vet Scand.

[6] Tyler JW, Besser TE, Wilson L, Hancock DD, Sanders S, Rea DE (1996). Evaluation of a whole blood glutaraldehyde coagulation test for the detection of failure of passive transfer in calves. J Vet Intern Med.

[7] Raboisson D, Trillat P, Cahuzac C (2016). Failure of passive immune transfer in calves: a meta-analysis on the consequences and assessment of the economic impact. PLoS ONE.

[8] Barry J, Bokkers EAM, Berry DP, de Boer IJM, McClure J, Kennedy E (2019). Associations between colostrum management, passive immunity, calf-related hygiene practices, and rates of mortality in preweaning dairy calves. J Dairy Sci.

[9] Beam AL, Lombard JE, Kopral CA, Garber LP, Winter AL, Hicks JA (2009). Prevalence of failure of passive transfer of immunity in newborn heifer calves and associated management practices on US dairy operations. J Dairy Sci.

[10] Cuttance EL, Mason WA, Laven RA, Phyn CVC (2018). The relationship between failure of passive transfer and mortality, farmer-recorded animal health events and body weights of calves from birth until 12 months of age on pasture-based, seasonal calving dairy farms in New Zealand. Vet J.

[11] Jaster EH (2005). Evaluation of quality, quantity, and timing of colostrum feeding on immunoglobulin G1 absorption in Jersey calves. J Dairy Sci.

[12] Morinz DE, McCoy GC, Hurley WL (1997). Effects of quality, quantity, and timing of colostrum feeding and addition of a dried colostrum supplement on immunoglobulin G1 absorption in Holstein bull calves. J Dairy Sci.

[13] Reschke C, Schelling E, Michel A, Remy-Wohlfender F, Meylan M (2017). Factors associated with colostrum quality and effects on serum gamma globulin concentrations of calves in swiss dairy herds. J Vet Intern Med.

[14] Hechenberger N, Lichtmannsperger K, Klein-Jöbstl D, Tichy A, Wittek T (2023). Assessment of herd, calf, and colostrum management practices on austrian dairy farms using a scoring system. Animals.

[15] McGuirk SM, Collins M (2004). Managing the production, storage, and delivery of colostrum. Vet Clin N Am Food Anim Pract.

[16] Buczinski S, Vandeweerd JM (2016). Diagnostic accuracy of refractometry for assessing bovine colostrum quality: a systematic review and meta-analysis. J Dairy Sci.

[17] Stott GH, Marx DB, Menefee BE, Nightengale GT (1979). Colostral immunoglobulin transfer in claves II. The rate of absorption. J Dairy Sci.

[18] Gelsinger SL, Jones CM, Heinrichs AJ (2015). Effect of colostrum heat treatment and bacterial population on immunoglobulin G absorption and health of neonatal calves. J Dairy Sci.

[19] Klein-Jöbstl D, Arnholdt T, Sturmlechner F, Iwersen M, Drillich M (2015). Results of an online questionnaire to survey calf management practices on dairy cattle breeding farms in Austria and to estimate differences in disease incidences depending on farm structure and management practices. Acta Vet Scand.

[20] Geiger AJ (2020). Colostrum: back to basics with immunoglobulins. J Anim Sci.

[21] Lombard J, Urie N, Garry F, James R, Maas J, Sterner K (2020). Consensus recommendations on calf- and herd-level passive immunity in dairy calves in the United States. J Dairy Sci.

[22] Weaver DM, Tyler JW, VanMetre DC, Hostetler DE, Barrington GM (2000). Passive transfer of colostral immunoglobulins in calves. J Vet Intern Med.

[23] Deelen SM, Ollivett TL, Haines DM, Leslie KE (2014). Evaluation of a Brix refractometer to estimate serum immunoglobulin G concentration in neonatal dairy calves. J Dairy Sci.

[24] Elsohaby I, McClure JT, Keefe GP (2015). Evaluation of digital and optical refractometers for assessing failure of transfer of passive immunity in dairy calves. J Vet Intern Med.

[25] Renaud DL, Duffield TF, LeBlanc SJ, Kelton DF (2018). Short communication. Validation of methods for practically evaluating failed passive transfer of immunity in calves arriving at a veal facility. J Dairy Sci.

[26] Zakian A, Nouri M, Rasooli A, Ghorbanpour M, Constable PD, Mohammad-Sadegh M (2018). Evaluation of 5 methods for diagnosing failure of passive transfer in 160 holstein calves. Vet Clin Pathol.

[27] Elsohaby I, McClure JT, Waite LA, Cameron M, Heider LC, Keefe GP (2019). Using serum and plasma samples to assess failure of transfer of passive immunity in dairy calves. J Dairy Sci.

[28] Sutter F, Rauch E, Erhard M, Sargent R, Weber C, Heuwieser W (2020). Evaluation of different analytical methods to assess failure of passive transfer in neonatal calves. J Dairy Sci.

[29] Gamsjäger L, Elsohaby I, Pearson JM, Levy M, Pajor EA, Windeyer MC (2021). Evaluation of 3 refractometers to determine transfer of passive immunity in neonatal beef calves. J Vet Intern Med.

[30] Hare KS, Pletts S, Pyo J, Haines D, Guan LL, Steele M (2020). Feeding colostrum or a 1:1 colostrum:whole milk mixture for 3 days after birth increases serum immunoglobulin G and apparent immunoglobulin G persistency in Holstein bulls. J Dairy Sci.

[31] Delhez P, Meurette E, Knapp E, Theron L, Daube G, Rao AS (2021). Assessment of a rapid semi-quantitative immunochromatographic test for the evaluation of transfer of passive immunity in calves. Animals.

[32] Drikic M, Windeyer C, Olsen S, Fu Y, Doepel L, de Buck J (2018). Determining the IgG concentrations in bovine colostrum and calf sera with a novel enzymatic assay. J Anim Sci Biotechno.

[33] Elsohaby I, Keefe GP (2015). Preliminary validation of a calf-side test for diagnosis of failure of transfer of passive immunity in dairy calves. J Dairy Sci.

[34] Stilwell G, Carvalho RC (2011). Clinical outcome of calves with failure of passive transfer as diagnosed by a commercially available IgG quick test kit. Can Vet J.

[35] Lichtmannsperger K, Hartsleben C, Spöcker M, Hechenberger N, Tichy A, Wittek T (2023). Factors associated with colostrum quality, the failure of transfer of passive immunity, and the impact on calf health in the first three weeks of life. Animals.

[36] Landis JR, Koch GG (1977). The measurement of observer agreement for categorical data. Biometrics.

[37] Bartens MC, Drillich M, Rychli K, Iwersen M, Arnholdt T, Meyer L (2016). Assessment of different methods to estimate bovine colostrum quality on farm. N Z Vet J.

[38] George JW (2001). The usefulness and limitations of hand-held refractometers in veterinary laboratory medicine: an historical and technical review. Vet Clin Pathol.

[39] Dubin S, Hunt P (1978). Effect of anticoagulants and glucose on refractometric estimation of protein in canine and rabbit plasma. Lab Anim Sci.

[40] Abuelo A, Cullens F, Hanes A, Brester JL (2021). Impact of 2 versus 1 colostrum meals on failure of transfer of passive immunity, pre-weaning morbidity and mortality, and performance of dairy calves in a large dairy herd. Animals.

